# Evaluating the Effect of Pressure-Controlled Versus Volume-Controlled Ventilation Modes on Intraoperative Bleeding in Rhinoplasty: A Randomized Clinical Trial

**DOI:** 10.5812/aapm-151582

**Published:** 2024-10-29

**Authors:** Behrooz Zaman, Masood Mohseni, Samad Noorizad, Soudabeh Jalali Motlagh, Taymaz Amiraslani, Monal Sayyahi

**Affiliations:** 1 School of Medicine, Iran University of Medical Sciences, Tehran, Iran; 2 Pain Research Center, School of Medicine, Iran University of Medical Sciences, Tehran, Iran; 3 Department of Anesthesiology and Pain, School of Medicine, Iran University of Medical Sciences, Tehran, Iran

**Keywords:** Rhinoplasty, Intraoperative Bleeding, Ventilation Mode, Hemodynamics

## Abstract

**Background:**

Intraoperative bleeding is one of the major challenges in rhinoplasty.

**Objectives:**

This study aimed to evaluate the effect of pressure-controlled ventilation (PCV) versus volume-controlled ventilation (VCV) modes on intraoperative bleeding during rhinoplasty.

**Methods:**

In a double-blinded randomized clinical trial, 58 candidates for rhinoplasty were randomly assigned to the PCV or VCV groups. Anesthesia was induced and maintained using the same total intravenous anesthesia (TIVA) method in both groups. The amount of bleeding was assessed by counting blood-soaked gauze and measuring the content of the suctioned fluid. Additionally, bleeding in the surgical field was assessed by the surgeon using the Boezaart criterion.

**Results:**

The mean intraoperative bleeding volume was 30 ± 45 mL in the PCV group and 100 ± 120 mL in the VCV group (P < 0.001). According to logistic regression analysis, the odds of experiencing moderately severe or severe bleeding in the VCV group were 5.4 times higher than in the PCV group. After adjusting for confounding variables, the odds ratio increased to 26.8 (95% CI = 1.2, 59.3).

**Conclusions:**

The results of the study suggest that the pressure-controlled mode may lead to lower intraoperative bleeding compared to the volume-controlled mode. The decrease in peak airway pressure is likely a contributing factor to this observation.

## 1. Background

One significant concern in rhinoplasty is intraoperative bleeding, which can profoundly impact both the outcome and the overall patient experience (1). The nasal mucosa contains numerous blood vessels, and the rich blood flow makes it susceptible to both spontaneous and traumatic bleeding. The presence of a common capillary network and abundant blood supply can lead to excessive bleeding during surgery (2).

Severe intraoperative bleeding presents several challenges during rhinoplasty. It can impair the surgeon’s visibility, making it difficult to assess and manipulate nasal structures accurately (2). This, in turn, can lead to suboptimal surgical outcomes and may necessitate additional corrective procedures. Furthermore, severe bleeding increases the risk of complications, including hematoma formation, postoperative infections, and prolonged recovery periods (1, 3, 4).

Existing evidence suggests that several factors influence intraoperative bleeding during rhinoplasty (5, 6). These factors include the patient's positioning, certain medications, the type of analgesia used, and the ventilation mode employed (3, 7-9). Improving intraoperative mechanical ventilation plays a crucial role in reducing various complications (10).

The type of ventilation, one of the key factors affecting intraoperative bleeding, influences the amount of bleeding by altering intrathoracic pressure, venous blood flow, and blood pressure (11, 12). The choice of ventilation mode should be made according to the patient's condition, the type of surgery, and other related factors (13). Additionally, careful monitoring of the patient's hemodynamic status and adjusting ventilator parameters during the operation are essential for optimal outcomes (14).

Notably, previous studies have shown that pressure-controlled ventilation (PCV) modes offer advantages over volume-controlled ventilation (VCV) in reducing intraoperative bleeding and improving surgical outcomes (11, 15-17). However, most of these studies have primarily focused on supine and lower limb surgeries (11, 15-17), with limited data available on the impact of VCV and PCV modes in surgeries such as rhinoplasty.

## 2. Objectives

The present study aims to address this gap in the literature by comparing the effects of VCV and PCV on intraoperative bleeding in patients undergoing rhinoplasty surgery.

## 3. Methods

### 3.1. Study Design

The current study employed a randomized, double-blind, parallel-group trial design. It was conducted at Hazrat-e Fatemeh Hospital, Iran University of Medical Sciences, between 2021 and 2022.

### 3.2. Ethics Approval

The study received ethical approval from the Iran University of Medical Sciences Ethics and Review Board, with the assigned ethics code IR.IUMS.FMD.REC.1400.517. Before participation, all patients were fully informed about the study protocol, and informed consent was obtained from each participant. The trial was registered at the Iran Clinical Trial Registry, with the registry code IRCT20101026005026N13.

### 3.3. Inclusion and Exclusion Criteria

The study included elective cases referred to the Plastic and Reconstructive Surgery Center for rhinoplasty surgery. Participants were required to be between 18 and 50 years old, with a physical status of American Society of Anesthesiologists (ASA) classification I or II. Exclusion criteria included patients with a Body Mass Index (BMI) over 35, coagulation disorders, heart disease, lung disease, use of anticoagulant and antiplatelet drugs, or herbal medications that could affect the coagulation system. Patients with any of these conditions were excluded from the study.

### 3.4. Randomization and Concealment

Patients were randomly assigned to either PCV or VCV modes using a table of random numbers. To ensure random allocation, the randomization order was generated (random sequence generation). Researchers first determined the order in which the numbers in the table would be read (e.g., up, down, left, or right). Then, a number from the table was selected randomly, and the subsequent numbers were chosen based on the predetermined direction. Even numbers were assigned to the PCV intervention, and odd numbers were assigned to the VCV intervention, with a 1:1 allocation ratio.

The assigned group was recorded on paper and placed in a sealed envelope. The envelopes containing the assigned interventions were coded with 8-digit codes (without the intervention name) and provided to the surgical team. After each patient’s enrollment, the anesthesiologist was instructed to open a specific code and apply the corresponding ventilation mode. Throughout the study, both the patients and the surgeon were blinded to the assigned intervention, ensuring blinding. Since the intervention names were not disclosed and were coded, the type of group could not be distinguished.

### 3.5. Sample Size and Sampling Method

The overall sample size for the study was determined to be 60 patients. Participants were enrolled using a simple random sampling method from among eligible patients who had provided informed consent.

To calculate the sample size, the following equation was used:


$$n=\frac{{\left(Z_{1-\frac{\alpha }{2}}+Z_{1-\frac{\beta }{2}}\right)}^{2}\;\;({SD}_{1}^{2}+{SD}_{2}^{2})}{d^{2}}$$

### 3.6. Sample Size and Sampling Method

The overall sample size for the study was determined to be 60 patients. Participants were enrolled using a simple random sampling method from among eligible patients who had provided informed consent.

To calculate the sample size, the following equation was used:

In this equation, α was set to 0.05 (corresponding to a significance level of 5%), and the power of the study was set at 80%. The values of SD1 and SD2 were determined to be 6.84 and 4.7, respectively. The difference between the compared groups was extracted from Moningi et al. (18), and it was considered to be 2.6 (the average difference in bleeding = 2.6). By plugging these values into the equation, the sample size for each group was calculated to be 30.

### 3.7. Intervention and Data Collection

After obtaining the appropriate venous line and initiating intravenous fluid infusion, standard monitoring connections were established, and clinical and demographic information of the patients was recorded in the checklist.

Premedication included the administration of midazolam at a dose of 20 µg/kg and fentanyl at a dose of 3 µg/kg of body weight (BW). Anesthesia induction was performed using lidocaine at a dose of 1 mg, propofol at a dose of 2 mg, and atracurium at a dose of 0.5 mg/kg of ideal BW. To maintain anesthesia, propofol was infused at a rate of 100 µg/kg/min, and remifentanil was administered at a dose of 0.1 to 0.2 µg/kg/min, aiming for a mean arterial pressure (MAP) of 60 - 65 mmHg (MAP = SBP + 2 (DBP)/3).

During the surgery, atracurium was injected at a dose of 0.2 mg/kg every 45 minutes. Additionally, dexamethasone was prescribed at a dose of 8 mg and ondansetron at a dose of 4 mg for each patient.

Patients were intubated using a spiral tube and placed on mechanical ventilation. In one group, patients underwent VCV with a tidal volume of 7 mL/kg BW, a respiratory rate of 10 breaths per minute, and an inhalation-to-exhalation ratio of 1:2. In the second group, patients received PCV with a pressure of 15 cmH_2_O and a respiratory rate of 10 breaths per minute. The pressure was gradually adjusted to achieve an end-tidal carbon dioxide (EtCO_2_) level of 35 - 40 mmHg.

### 3.8. Outcome Assessment

All patients underwent rhinoplasty surgery, performed by the same surgeon. The primary outcome of the study, the amount of bleeding, was assessed by weighing blood-soaked gauze (electronic balance, model: WT100001X, Readability: 0.1 g) and measuring the content of suctioned blood. To measure the blood inside the suction bottle and soaked gauze, a nurse anesthetist, who was unaware of the patient's ventilation mode, was asked to assist. This method has been used in several studies to measure blood loss during surgery (19-21).

The surgeon's satisfaction level was evaluated using a Likert Scale, and bleeding in the surgical field was assessed using the Boezaart criterion at the end of the operation, with a questionnaire administered to the surgeon (22). Mean arterial blood pressure, heart rate, and maximum airway pressure were monitored and recorded. In the event of bleeding exceeding 100 cc in the total volume of blood in the suction and gauze, tranexamic acid was administered at a dose of 10 - 15 mg/kg.

### 3.9. Statistical Analysis

Continuous variables were described using the mean and standard deviation (SD). For variables with a non-parametric distribution, the median and interquartile range (IQR) were provided. Categorical variables were described using frequency counts and percentages.

To assess the mean difference between the compared groups, an independent *t*-test was conducted. Additionally, the bleeding variable was categorized, with moderately severe and severe bleeding considered as the outcome of interest. Logistic regression was then performed to calculate the odds of severe bleeding in the VCV group compared to the PCV group. Age, gender, and BMI were included in the model as potential confounders. All statistical analyses were performed using Stata software (Version 17.0, College Station, Texas, USA). A significance level of P < 0.05 was used to determine statistical significance.

## 4. Results

**Figure 1.**
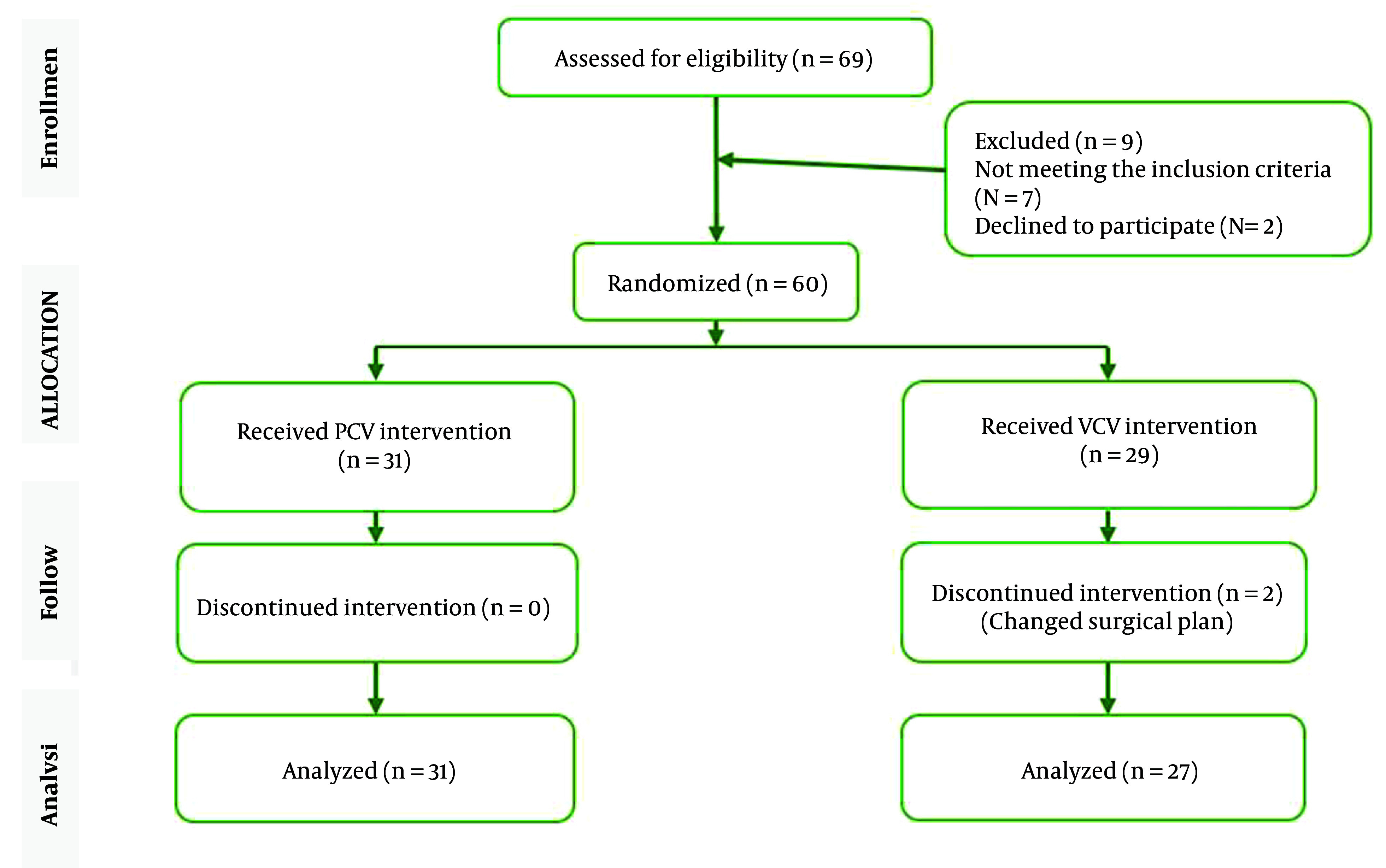
CONSORT diagram

The study was conducted on 58 eligible patients who underwent rhinoplasty surgery using either PCV or VCV modes in 2022. Initially, 60 patients were enrolled in the study, and 58 of them completed the written informed consent process. One patient did not receive the allocated intervention and was transferred to the comparison group. Among the participants, 31 patients were assigned to the PCV group, while 27 were assigned to the VCV group (Figure 1). 

The mean age of the study participants in the PCV and VCV groups was 34.6 ± 9.1 and 28.5 ± 6.9 years, respectively. The observed difference in age between the two groups was statistically significant (P-value < 0.001). In terms of gender, the proportion of males in the PCV group was 12.9%, compared to 29.6% in the VCV group, which was not statistically significant (P-value = 0.061) (Table 1). 

**Table 1.** Study Participants’ Baseline Characteristics in Pressure-Controlled and Volume-Controlled Ventilation Modes ^a^

Characteristics	PCV Group (n = 31)	VCV Group (n = 27)	P-Value
**Age**	34.6 ± 9.1	28.5 ± 6.9	0.001
**Male gender**	4 (12.9)	8 (29.6)	0.061
**BMI**	24.1 ± 2.9	23.8 ± 3.7	0.812

Abbreviations: PCV, pressure-controlled ventilation; VCV, volume-controlled ventilation; BMI, Body Mass Index.

^a^ Values are expressed as mean ± SD or No. (%).

Table 2 presents a comparison of intraoperative indices between the PCV and VCV groups. According to the table, there was no statistically significant difference in the average heart rate, mean arterial pressure, and average EtCO_2_ between the groups during the surgery (P-value > 0.05). However, there was a statistically significant difference in the average peak airway pressure, with values of 16.1 ± 1.6 cm H_2_O in the VCV group and 14.0 ± 1.4 cm H_2_O in the PCV group (P-value < 0.001).

**Table 2.** Comparison of Intraoperative Features of the Study Participants in Volume-Controlled Ventilation and Pressure-Controlled Ventilation Groups

Variables	PCV Group	VCV Group	P-Value
**Heart rate, beats.min** ^**-1**^	69.0 (9.6)	68.8 (8.7)	0.932
**Mean arterial pressure **	69.2 (6.4)	69.4 (6.1)	0.931
**EtCO**_**2**_^**a**^ **, mmHg**	35.3 (2.3)	34.1 (2.8)	0.080
**Pick airway pressure, Cm H**_**2**_ **O ** ^**b**^	14.0 (1.4)	16.1 (1.6)	0.001
**Blood loss, mL**	30.0 (45.0)	100 (120.0)	0.001

Abbreviations: PCV, pressure-controlled ventilation; VCV, volume-controlled ventilation.

^a^ End-tidal carbon dioxide.

^b^ Centimeter of water.

**Figure 2.**
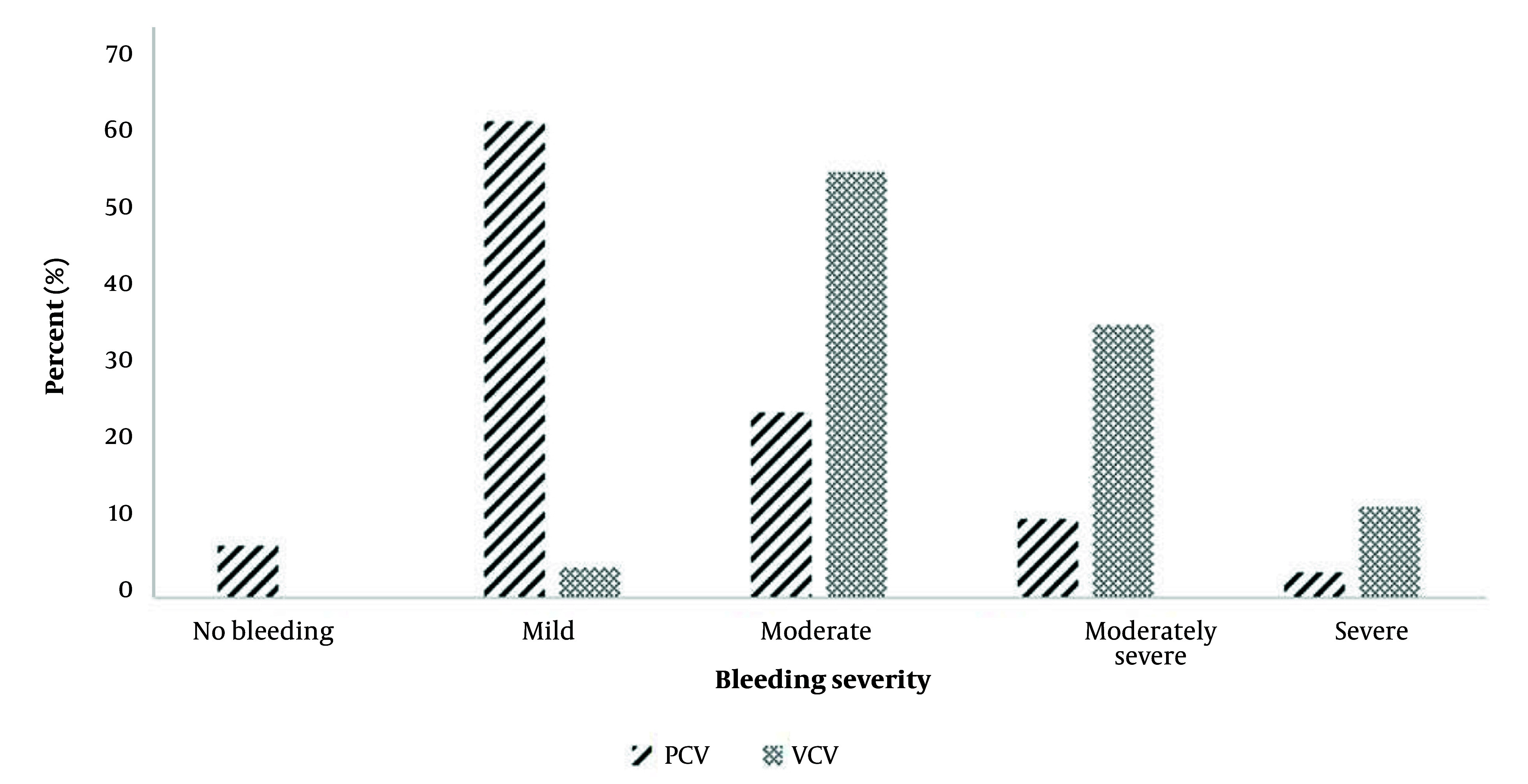
Intra-operative bleeding severity in pressure-controlled ventilation (PCV) and volume-controlled ventilation (VCV) groups according to Boezaart criterion

The median intraoperative bleeding volume was 30.0 mL for patients in the PCV group, while it was 100 mL in the VCV group (P-value < 0.001). We also compared the level of intraoperative bleeding using the Boezaart scale between the PCV and VCV groups. In the VCV group, the proportion of moderately severe and severe bleeding was 33.3% and 11.1%, respectively. In contrast, in the PCV group, 9.7% of patients experienced moderately severe bleeding, and the proportion of severe bleeding was 3.2% (P-value < 0.001) (Figure 2). 

**Figure 3.**
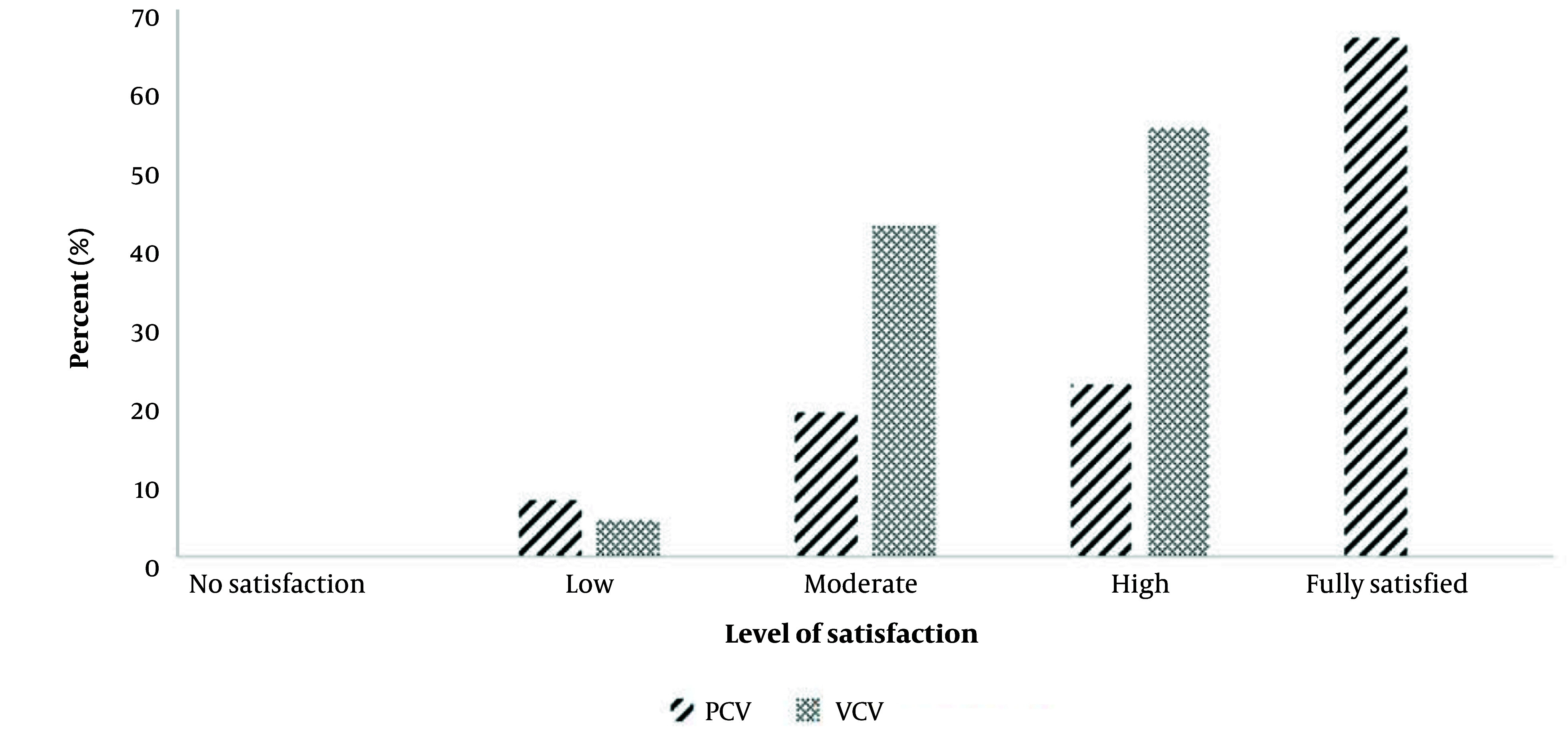
Surgeon’s satisfaction regarding intraoperative bleeding using the Likert Scale.

According to logistic regression analysis, the odds of experiencing moderately severe or severe bleeding in the VCV group were 5.4 times higher than in the PCV group. After adjusting for confounding variables, the odds ratio increased to 26.8 (95% CI = 1.2, 59.3) (Table 3). The level of surgeon satisfaction regarding intraoperative bleeding was significantly higher in the PCV group compared to the VCV group (P-value < 0.001) (Figure 3). 

**Table 3.** The Association Between Ventilation Mode and Severe Intraoperative Bleeding in Patients Who Underwent Rhinoplasty Surgery ^a^

Groups	No. of Event	Simple Model		Multiple Model	
		OR (95% CI)	P-Value	OR (95% CI)	P-Value
**PCV**	4 (12.9)	Reference		Reference	
**VCV**	12 (44.4)	5.4 (1.4,19.7)	0.011	26.8 (1.2, 59.3)	0.001

Abbreviations: PCV, pressure-controlled ventilation; VCV, volume-controlled ventilation; OR, odds ratio; CI, confidence interval.

^a^ Values are presented as No. (%).

Tranexamic acid was administered to 8 patients in the VCV group, in accordance with the research protocol, and no complications were reported in either group.

## 5. Discussion

Intraoperative bleeding is a crucial factor that directly influences the surgical outcome of rhinoplasty (23, 24). Over the past decades, various methods, such as patient head positioning and medicinal interventions like epinephrine injection at the surgical site, have been utilized to reduce blood pressure and minimize bleeding. These approaches have shown promising results in reducing bleeding during rhinoplasty surgery (8, 22, 25, 26).

Ventilation modes represent another approach to controlling airway pressure and potentially decreasing bleeding during rhinoplasty surgery. Creating suitable conditions for ease of venous return, especially from the head and neck, and controlling intrathoracic pressure are critical factors in reducing bleeding and ensuring a clear surgical field during rhinoplasty. During surgical procedures above the trunk, maintaining the proper body position, ensuring adequate head and neck positioning, and controlling the number and volume of breaths can significantly contribute to enhancing venous return and reducing bleeding.

Previous studies have explored the impact of different ventilation modes on reducing bleeding during spine and abdominal surgeries (27). The current study aimed to investigate and compare the effects of PCV and VCV modes on the amount of bleeding during rhinoplasty surgery. The comparison of intraoperative bleeding between the VCV and PCV groups revealed that patients in the VCV group experienced significantly higher levels of bleeding, which ultimately resulted in a lower satisfaction level for the surgeon. Additionally, when comparing peak airway pressure during the surgery, it was observed that patients in the VCV group had higher peak airway pressure values.

Our findings regarding higher intraoperative bleeding were consistent with a previous study conducted by Kang et al. (11), which also demonstrated lower intraoperative bleeding in patients who underwent PCV mode ventilation for posterior lumbar interbody fusion surgery. Similar results were reported by El-Sayed et al. (15) during lumbar discectomy in the prone position, supporting the notion that PCV mode ventilation can significantly reduce bleeding during rhinoplasty surgery. Lower intraoperative bleeding may be attributed to a reduction in peak airway pressure. Several other studies have also indicated that PCV can significantly reduce peak airway pressure, which is consistent with our findings (17, 25).

In contrast, VCV mode provides continuous ventilation to maintain a set tidal volume regardless of peak airway pressure (28). This approach can result in higher peak inspiratory pressure (PIP) and increased pressure on the airways, which may contribute to intraoperative bleeding (28). Koh et al. suggest that the increase in peak airway pressure during VCV mode ventilation can lead to changes in bleeding (17). Patients in the VCV group have been associated with higher intraoperative bleeding when repositioned from a supine to a prone position (23).

Additionally, Han et al. conducted a meta-analysis and found that the reduction of airway and abdominal pressure in PCV mode is the main reason for lower intraoperative bleeding. They propose that airway pressure is a significant factor affecting cardiac compliance and increasing cardiac preload, both of which can impact intraoperative bleeding (16).

The lower PIP in patients using PCV can also be attributed to the specific characteristics of this ventilation method. Pressure-controlled ventilation mode involves a high initial flow rate during the early stages of inspiration, followed by a gradual decrease in flow as inspiration progresses (7, 29). This flow pattern leads to a decrease in breathing pressure and promotes a more even distribution of gases throughout the lungs. By employing this approach, PCV mode ensures a more controlled and gentle delivery of inspired gas, resulting in a reduced PIP compared to other ventilation modes (29).

This optimized flow rate throughout the inspiratory phase helps minimize peak pressure on the airways, which likely contributes to the observed decrease in intraoperative bleeding. Additionally, the uniform distribution of gases achieved through PCV mode improves ventilation and enhances gas exchange within the lungs. This can lead to improved oxygenation and efficient removal of carbon dioxide, potentially reducing the risk of complications and improving overall patient outcomes (29).

The present study is a unique research endeavor focused on patients undergoing rhinoplasty surgery, specifically investigating the effectiveness of pressure-controlled and VCV modes. It stands out as one of the few studies conducted in this population, contributing valuable insights to the existing literature in this field. To minimize confounding factors, all surgeries were performed by a single surgeon, thereby eliminating the potential influence of varying surgical skills on intraoperative bleeding. Additionally, both the patients and the surgeon were blinded to the treatment protocol and randomization, reducing the possibility of bias or interference in the treatment process.

While blood loss during surgery is a concern due to its potential complications, it may have a greater impact on rhinoplasty due to the limited surgical field. The surgeon's visibility and maneuverability can be affected, and excessive bleeding can prolong the operation time, increasing the risks and potentially altering the surgical outcomes. For this reason, in addition to measuring the amount of blood in the used gauze and aspirated blood, we also compared the surgeon's evaluation of the bleeding amount using the Boezaart criteria between the two groups.

Kundra et al. conducted a study on patients undergoing lumbar spine surgery, where they compared the amount of bleeding and disruption in the surgical field between two groups of patients under pressure-controlled and VCV modes (26). They collected feedback from both the assistant and the scrub nurse. Their results are consistent with the findings of the present study.

In our study, even a small amount of blood could significantly disrupt the surgical field, so we sought the surgeon's opinion for the subjective evaluation of bleeding. Additionally, we compared the level of surgeon satisfaction between the two groups using a Likert Scale, alongside estimating the bleeding in the surgical field.

However, it is important to acknowledge the limitations of the study when interpreting its findings. One notable limitation is the relatively small sample size, which can affect the generalizability and statistical power of the results. Additionally, non-adherence to the randomization protocol led to the withdrawal of some patients from the study, potentially introducing bias. Furthermore, conducting the study in a single center limits the external validity of the findings and raises questions about whether the results can be generalized to other clinical settings. Future studies conducted across multiple centers with larger sample sizes can provide a more comprehensive understanding of the effects of pressure-controlled and VCV modes in rhinoplasty surgery.

### 5.1. Conclusions

The results of the study suggest that PCV mode may lead to lower intraoperative bleeding compared to VCV mode. The reduction in peak airway pressure, achieved through PCV mode, is likely a contributing factor to this observation. Additionally, the unique flow pattern of PCV, which features a high initial flow rate followed by a gradual decrease, facilitates a reduction in breathing pressure and promotes a more uniform distribution of gases throughout the lungs.

This study provides valuable insights into the effectiveness of pressure-controlled and VCV modes in rhinoplasty surgery. The results support the potential benefits of PCV mode in reducing intraoperative bleeding. These findings can inform clinical practice and guide the selection of ventilation strategies for patients undergoing rhinoplasty surgery, ultimately contributing to improved patient outcomes and safety. Further research is needed to corroborate these findings and address the limitations identified in this study.

## Data Availability

The dataset presented in the study is available on request from the corresponding author during submission or after publication. The data are not publicly available due to privacy.

## Clinical Trial Registration Code:

IRCT20101026005026N13.
